# Microcalorimetry: A Novel Application to Measure In Vitro Phage Susceptibility of *Staphylococcus aureus* in Human Serum

**DOI:** 10.3390/v15010014

**Published:** 2022-12-20

**Authors:** Michèle M. Molendijk, My V. T. Phan, Lonneke G. M. Bode, Nikolas Strepis, Divyae K. Prasad, Nathalie Worp, David F. Nieuwenhuijse, Claudia M. E. Schapendonk, Bouke K. H. L. Boekema, Annelies Verbon, Marion P. G. Koopmans, Miranda de Graaf, Willem J. B. van Wamel

**Affiliations:** 1 Department Medical Microbiology and Infectious Diseases, Erasmus MC, 3015 Rotterdam, The Netherlands; 2 Department of Viroscience, Erasmus MC, 3015 Rotterdam, The Netherlands; 3 Medical Research Council/Uganda Virus Research Institute, London School of Hygiene & Tropical Medicine Uganda Research Unit, Entebbe P.O. Box 49, Uganda; 4 Association of Dutch Burn Centres, 1941 AJ Beverwijk, The Netherlands

**Keywords:** bacteriophage therapy, *Staphylococcus aureus*, microcalorimetry, MRSA, susceptibility testing, human serum

## Abstract

Infections involving antibiotic resistant *Staphylococcus aureus* (*S. aureus*) represent a major challenge to successful treatment. Further, although bacteriophages (phages) could be an alternative to antibiotics, there exists a lack of correlation in phage susceptibility results between conventional in vitro and in vivo assays. This discrepancy may hinder the potential implementation of bacteriophage therapy. In this study, the susceptibility of twelve *S. aureus* strains to three commercial phage cocktails and two single phages was assessed. These *S. aureus* strains (including ten clinical isolates, five of which were methicillin-resistant) were compared using four assays: the spot test, efficiency of plating (EOP), the optical density assay (all in culture media) and microcalorimetry in human serum. In the spot test, EOP and optical density assay, all cocktails and single phages lysed both methicillin susceptible and methicillin resistant *S. aureus* strains. However, there was an absence of phage-mediated lysis in high concentrations of human serum as measured using microcalorimetry. As this microcalorimetry-based assay more closely resembles in vivo conditions, we propose that microcalorimetry could be included as a useful addition to conventional assays, thereby facilitating more accurate predictions of the in vivo susceptibility of *S. aureus* to phages during phage selection for therapeutic purposes.

## 1. Introduction

*Staphylococcus aureus* (*S. aureus*) causes many types of infections, ranging from relatively harmless skin infections to life-threatening endocarditis. In addition, it is the leading cause of bloodstream infections in industrialized countries [1,2,3]. In Europe, infections involving methicillin resistant *S. aureus* (MRSA) are the second most reported cause of death involving antimicrobial resistant pathogens [4]. Further, the high prevalence of antibiotic resistant *S. aureus* infections means that there is a need for new therapeutics against this pathogen, particularly against MRSA. A possible alternative to the use of antibiotics are bacteriophages (phages), which are viruses that can infect and lyse bacteria [5,6,7,8]. However, phages are generally very specific for individual strains of bacteria, meaning that the susceptibility of an infecting bacteria should be determined before patient treatment with phages begins. Currently however, there is a lack of standardized methods determining phage susceptibility of bacteria. Importantly, results of currently used in vitro assays for determining phage susceptibility do not always correlate with in vivo phage susceptibility results [9,10], possibly as a result of the complex interactions between phages, bacteria and the human microenvironment—which in vitro conditions might not adequately capture. For example, the expression and glycosylation of surface molecules (such as wall teichoic acid (WTA)) of *S. aureus* can be influenced by external environmental conditions [11,12,13,14,15]. As WTA may serve as a receptor for *S. aureus* phages, different environmental conditions may influence phage receptor availability and thus phage susceptibility [12,15]. Consequently, results from in vitro testing may lead to inadequate predictions of clinical outcomes; hence, hampering the advancement of phage therapy [9].

The use of animal models can aid in the translation of in vitro susceptibility results to in vivo phage efficacy in humans [16]. However, even though animal models allow investigation of bacteriophage efficacy under complex physiological conditions, they also suffer from drawbacks. For example, when it comes to studying *S. aureus* infections, the biggest limitation is differential host interaction observed between animal and human infections. In this respect, several host-specific virulence factors and immune evasion molecules are known to be responsible for the differences in host-pathogen interactions observed between *S. aureus* infections of mice and humans [17,18]. Additionally, when it comes to bacterial susceptibility testing of phages, animal experiments are time-consuming, come with substantial financial costs and involve multiple ethical considerations [19]. These factors mean that in vitro phage susceptibility testing assays will continue to remain important for phage susceptibility testing and phage selection prior to the administration of phage therapy.

In this publication, the authors used three in vitro assays to determine the phage susceptibility of a panel of *S. aureus* strains associated with several genomic clonal complexes (CCs). Strains within the CCs can differ in their glycosylation of the phage receptor WTA and in their restriction-modification (RM) systems. RM systems use specific methylation patterns to mark autologous DNA so that restriction enzymes can recognize this and destroy foreign DNA lacking these specific methylation patterns. This means that DNA of phages propagated in bacteria possessing one type of RM system can be recognized as foreign by bacteria possessing other types of RM systems, thereby limiting the range of bacterial strains that can be effectively treated by a phage propagated in a host of another CC [12,20,21]. Therefore, considering the potential influence of WTA and RM systems, the *S. aureus* panel used was chosen to represent the genetic variety of *S. aureus* CCs found in humans, while also displaying various clinically relevant antibiotic resistance profiles [22]. The phage susceptibility of our *S. aureus* panel was determined using three commercially available phage cocktails and two single phages, and was assessed in the spot test and optical density (OD) assay. These are regarded as the conventional assays for phage susceptibility testing prior to the selection of phages for phage therapy [9]. In addition, microcalorimetry (MC) was assessed as an additional tool to determine phage susceptibility in both bacterial culture media and human serum [23,24,25].

## 2. Materials and Methods

### 2.1. S. aureus Isolates and Phage Cocktails

*S. aureus* isolates used are listed in Table 1. The panel contains five MRSA isolates, including SaC042W and MW2 which are closely associated with community outbreaks in the US, Canada, Europe and Asia [26,27]. In addition to the recently isolated clinical strains, two laboratory strains were included: namely, RN4220 and R5. In contrast to the clinical strains, which were isolated from patients and hardly propagated since, the laboratory strains used have been passaged many times in laboratories around the world. RN4220 was chosen as it is widely used because it can accept foreign DNA due to a mutation in the restriction enzyme of its RM system [28]. The R5 strain was chosen because it is highly sensitive to phages and has historically been used for the propagation of phages for phage typing [29].

The commercial bacteriophage cocktails used were Georgian Pyofag cocktail (GPC) (Pharmex Group, Boryspil, Ukraine, batch #432118), Intestifag cocktail (INT) (Pharmex Group, Boryspil, Ukraine, batch #4311118) and Russian Pyobacteriophage cocktail (RPC) (NPO Microgen, Novgorod, Russia, batch #LS-000700). Multiple versions of these three cocktails are currently still commonly used in Russia and Georgia, and have been utilized both in animal models and clinical trials evaluating phage therapy [10,25,30,31,32,33].

**Table 1:** Panel of *S. aureus* isolates used in this study.

Isolate	Clonal Complex	MSSA/MRSA	Source	Accession Number	References
Mup15	CC15	MSSA	Nose, healthy carrier (The Netherlands, 1999–2001)	ERS12471228	[34,35]
Mup3199	CC25	MSSA	Nose, healthy carrier (The Netherlands, 1999–2001)	ERS12471229	[36]
Mup2723	CC30	MSSA	Nose, healthy carrier (The Netherlands, 1999–2001)	ERS12471230	[36]
Mup2396	CC45	MSSA	Nose, healthy carrier (The Netherlands, 1999–2001)	ERS12471231	[36]
SA2704	CC72	MSSA	Nose, healthy carrier (The Netherlands, 1999–2001)	ERS12471232	[36]
MW2 (USA400)	CC1	MRSA	Blood (USA, 1998)	NC_003923.1	[37]
Mu50	CC5	MRSA	VISA *, surgical wound (Japan, 1997)	BA000017.4	[38]
SAC042W (USA300)	CC8	MRSA	Skin abscess (USA, 2006–2008)	ERS12471233	[39,40]
M116	CC8	MRSA	Osteomyelitis (Indonesia, 2011)	ERS12471234	[41]
Rww146	CC398	MRSA	Live-stock associated clinical isolate	ERS12471235	[42,43]
RN4220	CC8	MSSA	Derivative of strain 8324-4	NZ_AFGU00000000.1	[28]
R5	CC30	MSSA	RIVM, Phage typing strain	ERS12471236	[29]

* Vancomycin intermediate *S. aureus.*

### 2.2. Single Phage Isolation and Production

Single phages were isolated from the RPC as described previously [44]. In short, a double layer assay was performed with the RPC using *S. aureus* strain R5 as the host. Plaques displaying different morphologies were picked and passaged on R5 until a single plaque morphology was obtained. Another double layer assay was performed to obtain full plate lysis to harvest the purified phage. The upper layer containing purified phages on the plates was scraped and suspended in SM buffer (100 mM NaCl, 8 mM MgSO_4_·7H_2_O and 1 M Tris-CI, pH 7.5), followed by centrifugation at 4000× *g* for 5 min to pellet any (bacterial) debris. The supernatant was stored at 4 °C until further use. To obtain high concentrations of each phage, *S. aureus* strain R5 was grown in 100 mL TSB and 20 µL of a single phage was added when the bacteria reached the exponential growth phase (OD_600_ = 0.3–0.6). The suspension of bacteria and phages was incubated overnight, shaking at 37 °C. After incubation, the phage lysate was centrifuged at 4000× *g* for 40 min at 4 °C. the supernatant was recovered and filtered using a 0.22 µm Whatman puradisc filter (Merck KGaA, Darmstadt, Germany). The filtrate was stored at 4 °C.

### 2.3. Next Generation Sequencing and Data Analysis

#### 2.3.1. *S. aureus* Isolates

MW2, Mu50 and RN4220 genomes were obtained from NCBI genomic repository (NC_003923.1, BA000017.4 and NZ_AFGU00000000.1, respectively). For the other isolates, DNA was isolated from freshly grown cultures using the Zymo Research Quick-DNA Fungal/Bacterial Miniprep Kit (Baseclear, Leiden, The Netherlands). Sequencing libraries were prepared from the extracted DNA using the Nextera DNA Flex Library Preparation Kit (Illumina, San Diego, CA, USA) and sequenced on an Illumina iSEQ 100 System (Illumina, San Diego, CA, USA), generating 150 bp paired-end reads. Then, reads were assembled using both CLC Genomics Workbench v20 (Qiagen, Hilden, Germany) and Unicycler v0.4 [45] with default parameters and analyzed using the available scheme in SeqSphere software v5.1.0 (Ridom, Munster, Germany). To assess the genomic diversity of the strains in our panel compared to global diversity, *S. aureus* genomes (*N* = 289 sequences) representing the current diversity of *S. aureus* were retrieved from Genbank using genomes that had been released in the year 2021 (1 January 2021–31 December 2021). The MLST and CC sequence type (ST) analyses were performed using SeqSphere+ v5.1 (Ridom, Münster, Germany). From the downloaded sequences, a maximum of fifteen genomic sequences per ST were randomly selected for further analysis, resulting in a dataset of 205 *S. aureus* genomes, including the genomic sequences of the twelve strains used in this study. All genomes were subjected to k-mer analysis using kSNP3 v1.1 [46] with default parameters and a k-mer size of 17. The generated maximum-likelihood tree from the core SNPs was uploaded in iTOL v6.5 [47].

For the twelve isolates used in this study, the presence of known phage-resistance genes, such as phage receptors and RM systems, described by Moller et al. (2021) was assessed using BLASTn [48]. The presence of known phage-resistance systems, such as Gabija and Thoeris, was examined using PADLOC, which is an online tool created for this purpose by Payne et al. [49,50,51].

#### 2.3.2. Phage Cocktails and Single Phages

To concentrate the single phages for sequencing PEG6000 (Merck KGaA, Darmstadt, Germany) was added. To enhance virus particle precipitation, the pH was adjusted to 4 using HCl [52]. After overnight incubation at 4 °C, the phages were centrifuged at 13,500× *g* for ninety minutes. The pellet containing the concentrated phages was dissolved in glycine buffer (glycine 3.75 g/L, NaCl 9 g/L, pH 9.5). Chloroform was added to lyse remaining (infected) bacterial cells and the sample was centrifuged for five minutes at 13,500× *g* to remove bacterial debris. After centrifugation, the upper phase containing the purified and concentrated phages was collected for sequencing. The phage cocktails were not concentrated or purified to conserve all phages present, but they were centrifuged for five minutes at 13,500× *g* to pellet bacterial debris and the supernatant was collected for sequencing. The concentrated single phages and phage cocktails were incubated separately with TURBO DNAse (ThermoFisher Scientific, Waltham, MA, USA) for 30 min at 37 °C to remove free nucleic acids. Next, total nucleic acids were extracted using the High Pure RNA Isolation Kit (cat #11828665001, Roche, Basel, Switzerland), of which the DNAse step was omitted to assure extraction of both RNA and DNA. RNA was transcribed into cDNA using random primers (Promega, Madison, WI, USA) and SuperScript IV (ThermoFisher Scientific, Waltham, MA, USA), followed by dsDNA synthesis using Klenow fragments (NEB). The resulting dsDNA was subjected to library preparation and Nanopore sequencing using the PCR Barcoding Kit SQK-PBK004 (Oxford Nanopore, Oxford, UK) according to the manufacturer’s instructions, on a FLO-MIN112 flowcell.

To obtain a general overview of the diversity of phages present in the three phage cocktails, a read-based taxonomic annotation was performed against all bacteriophage sequences available in Genbank, using BLASTn. The abundance of reads mapped to bacteriophages relative to the total amount of reads in the sample has been denoted in Supplementary Materials Table S1.

To specifically look at staphylococci infecting phages present in the phage cocktails, first the raw reads were demultiplexed using CD-HIT-DUP [53] and quality control was performed using fastp v0.12 [54]. Reads with length <75 nt and Phred score <10 were discarded. The resulting reads were de novo assembled using Canu v2.2, with an estimated genome size of 150 kb for single phages and high sensitivity settings [55]. Then, the contigs were mapped against five reference phage genomes representing the genera currently known to infect staphylococci: phage phiSA_BS2 (*Boashanvirus*, NC_047948.1), phage K (*Kayvirus*, KF766114.1), phiIBB-SEP1 (*Sepunavirus*, NC_041928.1), phage Remus (*Silviavirus*, MW546076.1), phage Twort (*Twortvirus*, MT151386.1). For the single phages, after assembly, the tail sheath protein genes (accession numbers OP352909 and OP352910) were aligned to the same five reference phage genomes. A maximum-likelihood nucleotide tree based on the tail sheath protein was constructed using IQTree v.1.6, with 1000 bootstraps [56,57] and visualized using FigTree (v1.4.) [58]. A sequence identity matrix of the partial genomes of RPCSa1 and RPCSa2, based on phage K (nt 40352 to nt 141284) as the reference genome, was created using BioEdit v7.2 [59].

### 2.4. Spot Test

Bacterial strains were grown overnight on Tryptic Soy Agar II plates with 5% sheep blood (TSA II) (BD, Franklin Lakes, NJ, USA). A single bacterial colony was incubated in Luria-Bertani (LB) broth (Merck KGaA, Darmstadt, Germany) at 37 °C and grown until the exponential phase (OD_600_ = 0.5 ± 0.2). Next, 200 µL of bacteria was added to 4 mL 0.35% LB agar (containing 1 M CaCl_2_ and 1 M MgSO_4_) and poured onto a 1.4% LB agar plate. Tenfold dilutions of the phage cocktails or single phages were prepared in SM buffer. When the 0.35% LB agar solidified, a 10 µL of serially diluted phage was pipetted onto the plate. Plates were incubated overnight at 37 °C and individual plaques were counted.

### 2.5. Optical Density (OD) Assay

*S. aureus* isolates were grown overnight on TSA II plates, suspended in TSB to OD_600_ = 0.5 (approx. 1 × 10^8^ colony forming units (cfu)/mL) and further diluted in TSB to achieve approximately 1 × 10^5^ cfu/mL. For the phage cocktails, threefold dilutions were made, starting at 2.4 × 10^6^ plaque-forming units (pfu)/mL (undiluted) in SM buffer. All phage concentrations were determined using *S. aureus* R5 and thus excluded phages infecting other bacteria. For the single phages, tenfold dilutions started at 3 × 10^8^ pfu/mL. Finally, 180 µL of the bacterial suspension was added to 20 µL of phage dilution in a flat bottom plate (Corning Inc., Corning, NY, USA). Bacterial suspension with 20 µL SM buffer containing gelatin was used as a positive control for bacterial growth and 180 µL TSB with 20 µL SM buffer as a negative control. Plates were incubated at 37 °C in the FLUOstar Omega (BMG Labtech, software v5.50), shaking at 100 revolutions per minute (rpm) before every measurement. Turbidity was measured as OD_600_ every 10 min for 24 h (Supplementary Materials Figure S1). After 24 h of incubation, the suspensions from each well were grown overnight at 37 °C on TSA II plates, colonies were counted and the cfu/mL was determined to validate the effect of phage dilution on bacterial growth. For clarity Graphpad Prism (GraphPad Software v8.4.1) was used to calculate the area under the curve (AUC) of the turbidity over time. The percentage of the AUC relative to the growth control (set at 100%) was visualized, allowing for better comparison of phage susceptibility of the twelve *S. aureus* strains used.

### 2.6. Efficiency of Plating (EOP)

To assess discrepancies between the spot test and OD assay, EOP was performed as previously described by Kropinski (2009) with some minor adjustments [60]. In short, bacterial strains were grown overnight on TSA II plates (BD, Franklin Lakes, NY, USA). A single bacterial colony was incubated in LB broth (Merck KGaA, Darmstadt, Germany) at 37 °C and grown until the exponential phase (OD_600_ = 0.5 ± 0.2). A ten- and hundred-times dilution of the phage cocktails or single phages were made in SM buffer. Next, 100 µL of either undiluted or diluted phages together with 200 µL of bacteria was added to 3 mL 0.35% LB agar (containing 1 M CaCl_2_ and 1 M MgSO_4_) and poured onto a 1.4% LB agar plate. Plates were incubated overnight at 37 °C and assessed for bacterial lysis.

### 2.7. Microcalorimetry (MC)

Microcalorimetry (MC) is used to determine the metabolic activity of bacteria which is depicted as heat flow (in µWatt) over time and can be used as a proxy for bacterial growth [61]. In contrary to the optical density assay, MC is not affected by aggregation of *S. aureus* in serum.

#### 2.7.1. Serum Used for Microcalorimetry

All serum was acquired from Sanquin blood supply in Amsterdam, The Netherlands. Serum was collected at Sanquin according to the European directives 2002/98/EC, 2004/33/EC and 2005/61/EC, and the General Data Protection Regulation (GDPR). In addition, Sanquin adheres to the Dutch law on acquirement of blood and blood components (BWBR0017977).

#### 2.7.2. Phage Cocktails

*S. aureus* isolates Mup15 and Mup2723 were grown overnight on TSA II plates. Colonies were suspended in phosphate-buffered saline (PBS) to an OD_600_ = 0.5 (~1 × 10^8^ cfu/mL). Rifampicin (Sigma Aldrich, Saint Louis, MO, USA) and flucloxacillin (Erasmus MC pharmacy) were diluted in heat-inactivated (HI) human serum pooled from four donors (Sanquin, Amsterdam, The Netherlands) to 40 µg/mL and 128 µg/mL, respectively. Bacteria were diluted in HI human serum, with or without antibiotics, to a final concentration 1 × 10^7^ cfu/mL. Either 10 µL undiluted phage cocktail (2.4 × 10^6^ pfu/mL) or PBS (growth control) was added to the bacteria in CalWel sterile inserts (SymCel, Solna, Sweden), resulting in a final serum concentration of 82% per well. The inserts were placed in titanium cups (SymCel, Solna, Sweden) and placed in the CalScreener (SymCel, Solna, Sweden) at 37 °C. Heat flow was measured for twenty hours. Data were analyzed with CalView (SymCel, Solna, Sweden). Graphpad Prism 5 (GraphPad Software) was used to calculate the area under the curve (AUC) of heat flow over time. The percentage of the AUC relative to the growth control (set at 100%) was visualized, allowing better comparison of phage susceptibility of the panel of twelve *S. aureus* strains.

#### 2.7.3. Single Phages

*S. aureus* isolates were grown overnight on TSA II plates and suspended in PBS to OD_600_ = 0.5 and further diluted to 3 × 10^7^ cfu/mL. Tenfold dilutions of the single phages were made in SM buffer with 1 × 10^10^ pfu/mL as the highest concentration. Ninety microliters of TSB, or human serum pooled from fifty donors (Sanquin, Amsterdam, The Netherlands), was added to 10 µL of bacterial dilution and 10 µL of phage dilution in CalWel sterile inserts (SymCel, Solna, Sweden), resulting in a final serum or TSB concentration of 82% per well. Bacteria in TSB, or bacteria in serum with 10 µL SM buffer without phages, were used as positive bacterial growth controls. TSB or serum with 10 µL SM and 10 µL PBS were used as a negative control. Heat flow was measured and analyzed as described above.

## 3. Results

### 3.1. Genetic Characteristics of Selected Clinical S. aureus Strains and Phage Cocktails

**Figure 1:**
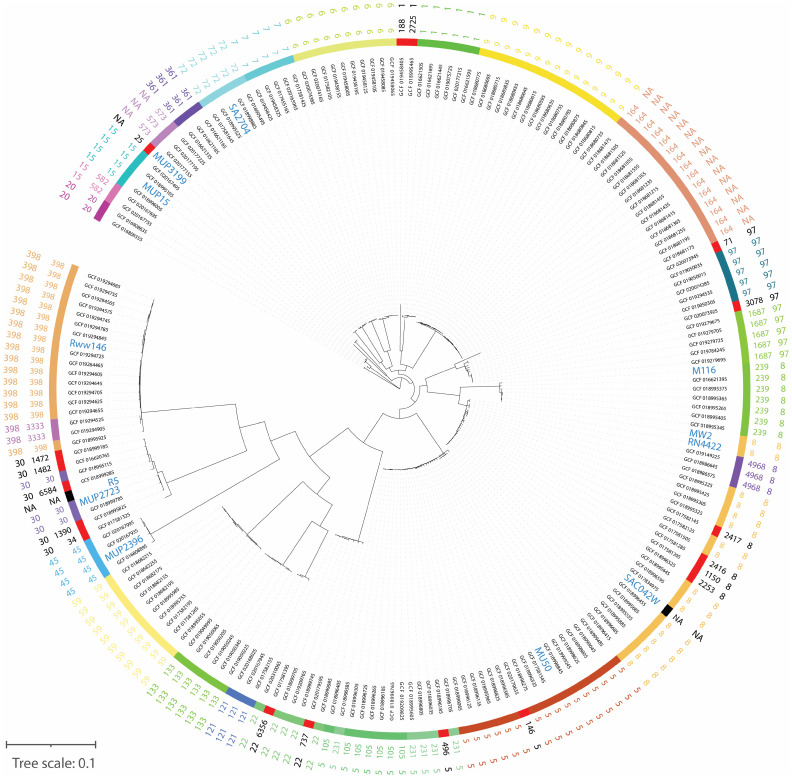
Circular visualization of the genomes of *S. aureus* strains in our panel compared to 205 *S. aureus* strains representing global genetic diversity. The maximum-likelihood tree in the figure describes the core SNP differences. From the inner to the outer circle, the first circle represents public genomes of *S. aureus* including the genomes from this study marked in blue and larger font, the second and third circles represents sequence type (ST) characterization visualized by color and text and the fourth circle represents CC information. Genomes without an ST and CC identification were symbolized with NA (Non-Available).

To establish the genetic diversity of our panel of *S. aureus* strains, all strains were subjected to whole genome sequencing. Multiple clonal complexes (CCs) among the *S. aureus* isolates were observed based on their core SNP differences (Figure 1). The twelve strains used in this study were widely distributed among different CCs and comprise four of five major human pathogenic lineages circulating globally [62].

Next, the presence of known phage-resistance genes and phage-resistance systems in the *S. aureus* strains of our panel were identified (Table 2) [48,63]. For most of the methicillin-sensitive *S. aureus* (MSSA) strains, at least one phage resistance gene or system was identified, except for Mup3199 which did not contain any of the phage resistance genes examined. Similarly, only one MRSA strain, SaC042W, lacked phage resistance genes. In both laboratory strains, phage defense genes were present.

**Figure 2:**
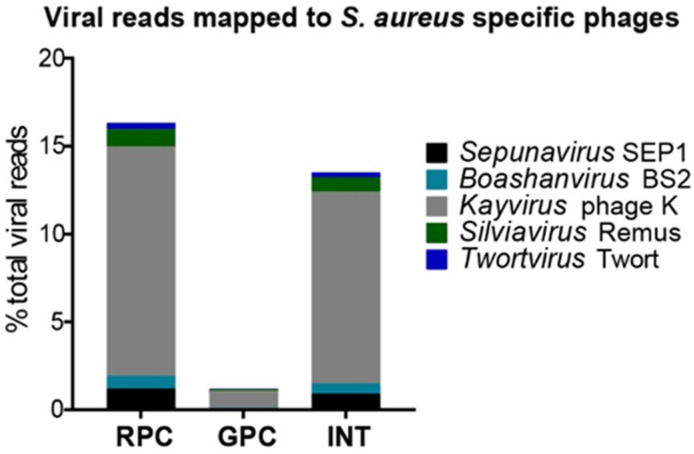
Presence of phages in three commercially available phage cocktails. Y-axis: Percentage of viral sequencing reads for the Russian- and Georgian-Pyofag cocktail (RPC and GPC, respectively) and the Intestifag cocktail (INT) mapped against the genome sequences of species types representing five genera that mostly comprise virulent phages against *S. aureus*.

All three phage cocktails are able to target not only *S. aureus* but also multiple other bacterial species. To examine the content of the three phage cocktails, next generation sequencing was used. Firstly, a general overview of phage diversity in the cocktails was obtained using BLASTn. This confirmed the presence of multiple bacteriophage genera that infect various bacterial hosts, including *S. aureus* (Supplementary Materials Table S1). Then, to specifically examine the presence of *S. aureus* infecting phages, reference-based mapping was performed. This showed that 16%, 1% and 13% for the RPC, GPC and INT cocktail, respectively, mapped to phages infecting *S. aureus.* In addition, the majority of reads mapped to *S. aureus* infecting phages were most similar to phage K—the species type for the *Kayvirus* genus in the subfamily *Twortvirinae* of the family *Herelleviridae* (Figure 2).

### 3.2. Determination of Phage Susceptibility Using Conventional In Vitro Assays

The susceptibility of the clinical and laboratory *S. aureus* strains to the three phage cocktails used was first assessed using the spot test. This test showed similar efficacy for both the RPC and GPC on the same strains, lysing five and six out of ten clinical strains, respectively (Table 3), despite the low percentage of reads mapping to phages infecting *Staphylococci* in the GPC (Figure 2). No major differences in phage susceptibility were observed between MSSA and MRSA strains for any of the three commercially available cocktails. Of these, INT exhibited the narrowest host range, lysing only four out of ten strains, of which three were MSSA strains. All three cocktails were effective against laboratory strain R5, however: only RPC and GPC lysed RN4220 (Table 3).

**Figure 3:**
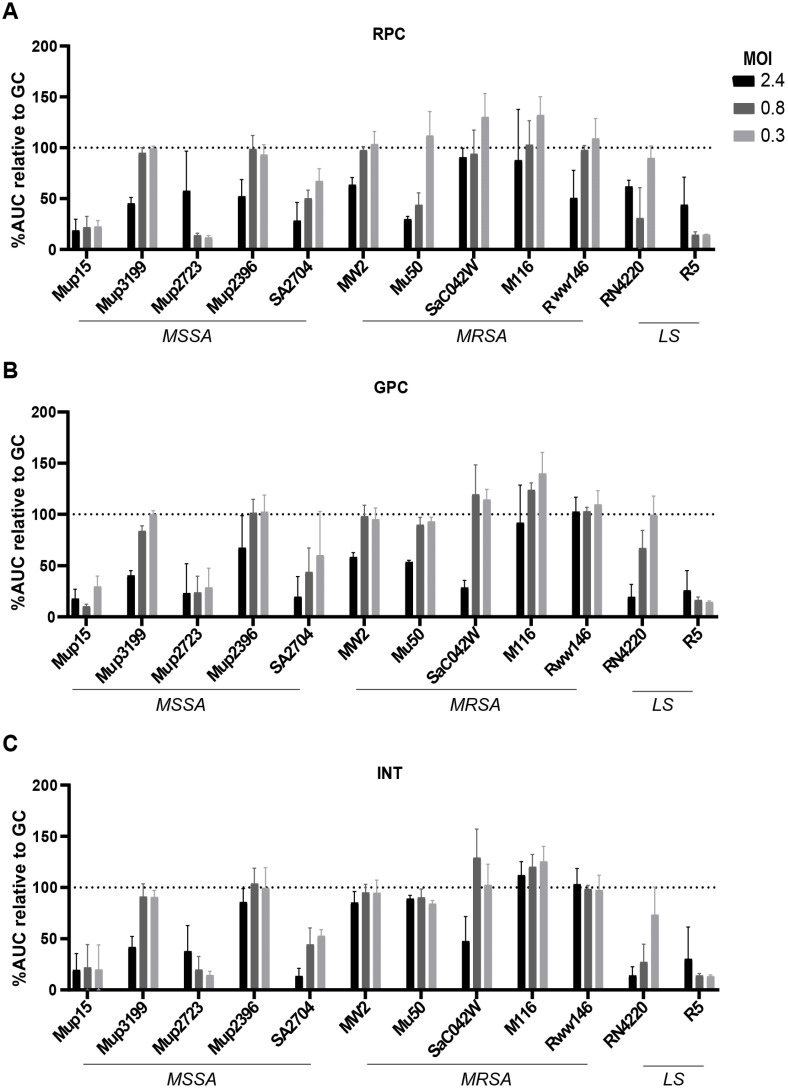
Susceptibility of *S. aureus* isolates to phage cocktails as determined by OD assay in TSB. The OD of five MSSA strains, five MRSA strains and two laboratory strains (LS) inoculated with the (**A**) RPC, (**B**) GPC and (**C**) INT at different multiplicity of infection (MOI) was measured for 24 h. The OD over time (24 h) is depicted as percentage of area under the curve (AUC) compared to the growth control, which was set at 100%. All conditions were repeated in three independent experiments.

Phage susceptibility was also determined in TSB using the OD assay (Figure 3). This assay allows for the use of planktonic bacteria and measures phage efficacy over time, providing insight into phage/bacteria dynamics important for monitoring phage resistance [9]. All ten clinical strains and the two laboratory strains were inoculated with different concentrations of phage cocktail and the OD was measured for 24 h. The area under the curve of the OD curves is shown as a percentage of the growth control, which was set at 100%. Overall, phage susceptibility varied between the five MSSA strains, with Mup15 and Mup2723 being most susceptible to all three cocktails (Figure 3A). In contrast, the OD of Mup2396 and Mup3199 only showed a decrease at the highest MOI despite the lack of known phage resistance genes present in the latter. Growth of the MRSA strains was only restricted at the highest MOI tested and MW2, Mu50 and Rww146 were completely insensitive to INT (Figure 3B). The laboratory strain R5 was very sensitive to the phage cocktails, even at an MOI of 0.003 (Supplementary Materials Figure S2), while RN4220 only showed a reduction in OD_600_ at the two highest MOIs (Figure 3C). However, the susceptibility to all three phage cocktails was similar per tested *S. aureus* strain.

Even though conventional assays showed lysis of most of the *S. aureus* strains in the panel, discrepancies between the two assays were also observed. For example, for multiple strains, including Mup3199, Mu50 and RN4220, lysis was observed in the OD assay but not in the spot test. It has been reported that the spot test could be less accurate than other (more labor-intensive) plate-based assays such as ‘efficiency of plating’ (EOP) [10,64]. Therefore, discrepancies observed between the spot test and OD assay were examined using EOP (Table 4). Even though EOP did match the results of the OD assay more often than the spot test, some discrepancies between both plate-based assays and OD assay still remained. Mu50, for example, was lysed by the RPC in the OD assay but not in the spot test or EOP (Table 4).

### 3.3. Susceptibility to Commercial Phage Cocktails in Human Serum Measured Using Microcalorimetry

**Figure 4:**
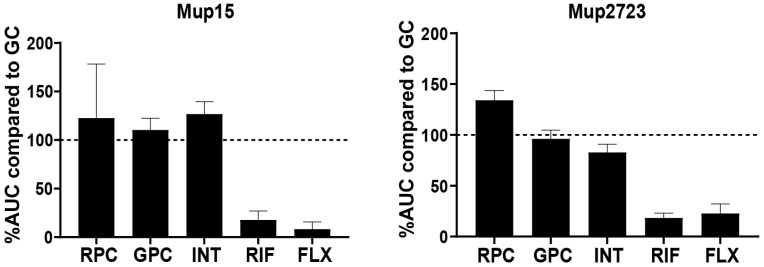
Metabolic activity of two phage sensitive strains in heat-inactivated human serum. Two phage-sensitive clinical *S. aureus* strains, Mup15 and Mup2723, were inoculated with phage cocktails RPC, GPC or INT an MOI 0.03 or with rifampicin (RIF; 40 µg/mL) or flucloxacillin (FLX; 128 µg/mL). As a positive control for bacterial growth, a control with PBS instead of phages or antibiotics was used. The heat flow was measured for 20 h and is shown as a percentage of the area under the curve (AUC) when compared to the positive growth control, which was set at 100%. All conditions were tested in three independent experiments.

More accurate results regarding phage susceptibility could potentially be achieved by resembling physiological conditions during bacterial infection, including phage susceptibility testing in the presence of human serum. However, the OD assay is not suitable to test phage susceptibility in this way, as serum components (such as fibrinogen and immune globulins) cause *S. aureus* to clump together in aggregates [65]. Microcalorimetry (MC) measures the metabolic activity of bacteria instead of optical density and is therefore not affected by *S. aureus* aggregation. However, *S. aureus* grows less efficiently in human serum compared to TSB, meaning that higher starting concentrations of *S. aureus* were required in order to measure sufficient metabolic activity of bacteria in human serum. With a higher starting concentration of bacteria, but not a higher concentration of the phage cocktails, the MOI was automatically reduced and therefore lower in the MC compare to the OD assay. For this protocol, we assessed the susceptibility of the two most phage-sensitive clinical *S. aureus* strains (Mup15 and Mup2723) to the phage cocktails in heat-inactivated human serum. As a control for bacterial cell death, the antibiotics rifampicin and flucloxacillin were used. While there was a clear effect of the antibiotics, the addition of the phage cocktails did not result in a decrease of metabolic activity at an MOI of 0.03 (Figure 4). Further, a higher MOI could not be tested as the phage cocktails were not concentrated due to the risk of losing or inactivating phages present in the original material [66]. Since the other clinical strains in the panel were not susceptible to the cocktails in TSB at MOI 0.03, they were not tested in human serum.

### 3.4. Susceptibility to Single Phages Using the Conventional In Vitro Assays

To test the effect of specific *S. aureus* phages, a total of eighteen phages were isolated from the RPC. The host range of these phages was determined using the spot test (data not shown). Two phages with a broad, but not identical, host range were selected: namely, RPCSa1 and RPCSa2 (Table 3). While *S. aureus* strain M116 was only lysed by the RPCSa1 phage, both phages lysed most other strains except for Mup2396, Mu50 and Rww146. Contrary to the RPC, from which these phages were isolated, both isolated phages were able to lyse Mup3199 and MW2.

**Figure 5:**
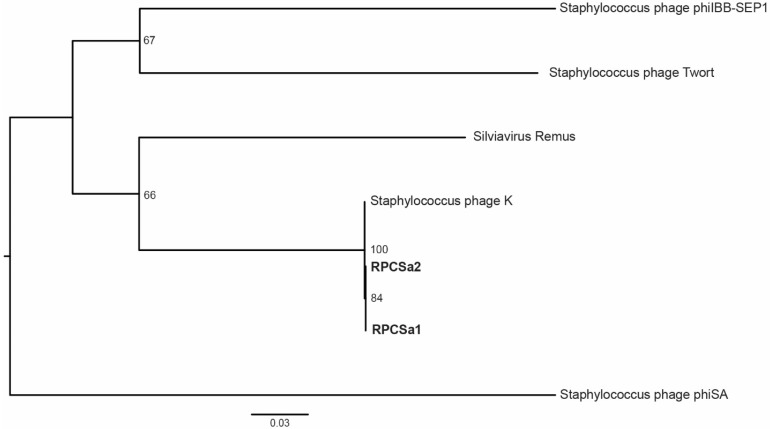
Phylogenetic tree of single phages and reference strains. A maximum-likelihood phylogenetic tree was inferred for the RPCSa1 and RPCSa2 and reference strains representing the diversity of staphylococci phages using the tail sheath protein gene. The tree was mid-point rooted and bootstrap values are shown at the nodes. Scale bars show the number of nucleotide substitutions per site.

Both phages RPCSa1 and RPCSa2 were subjected to next generation sequencing and comparison based on a maximum-likelihood tree of the tail sheath protein genes of reference strains of *S. aureus* infecting phages. Both phages were closest related to species type phage K representing the genus *Kayvirus* of subfamily *Twortvirinae* (Figure 5). In addition, a nucleotide identity matrix of their partial genomes showed 97.5% and 97.6% identity of RCPSa1 and RPCSa2 to phage K, respectively, and 99.8% identity to each other (data not shown).

**Figure 6:**
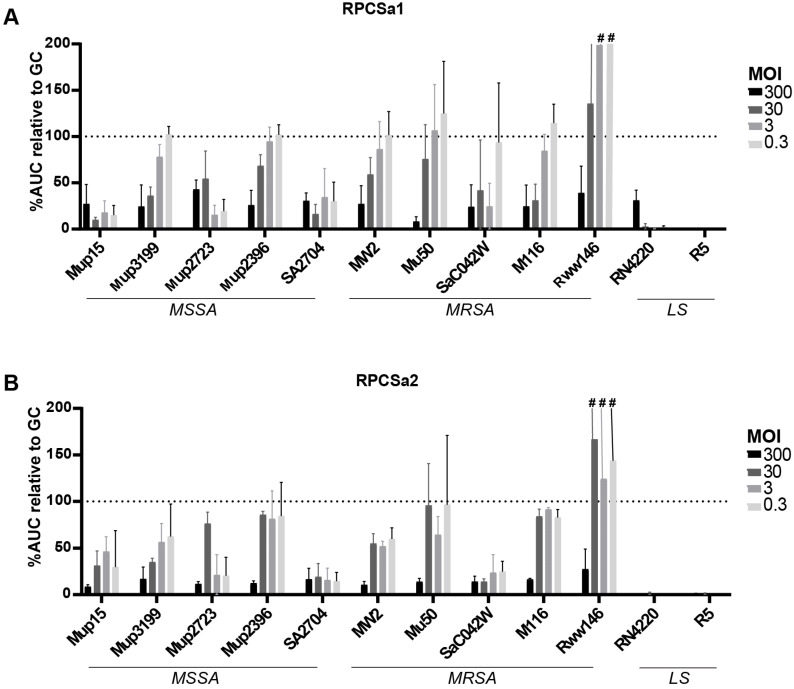
Susceptibility of *S. aureus* isolates to isolated single phages as determined by OD assay in TSB. The OD was measured for five MSSA strains, five MRSA strains and two laboratory strains (LS) inoculated with (**A**) RPCSa1 and (**B**) RPCSa2 at different MOIs. The OD over time (20 h) is shown as percentage of the area under the curve (AUC) compared to the growth control, which was set at 100%. ^#^ For clarity, values above 200% are not shown. All conditions were tested in three independent experiments.

The broad host range of the single phages was also shown using the OD assay (Figure 6). However, in contrast to the spot test, the OD assay showed lysis of all *S. aureus* strains by RPCSa1 and RPCSa2, including Mup2396, Mu50 and Rww146, but only at the highest MOI (Figure 6A,B).

### 3.5. Susceptibility to Single Phages in Human Serum Measured Using Microcalorimetry

**Figure 7:**
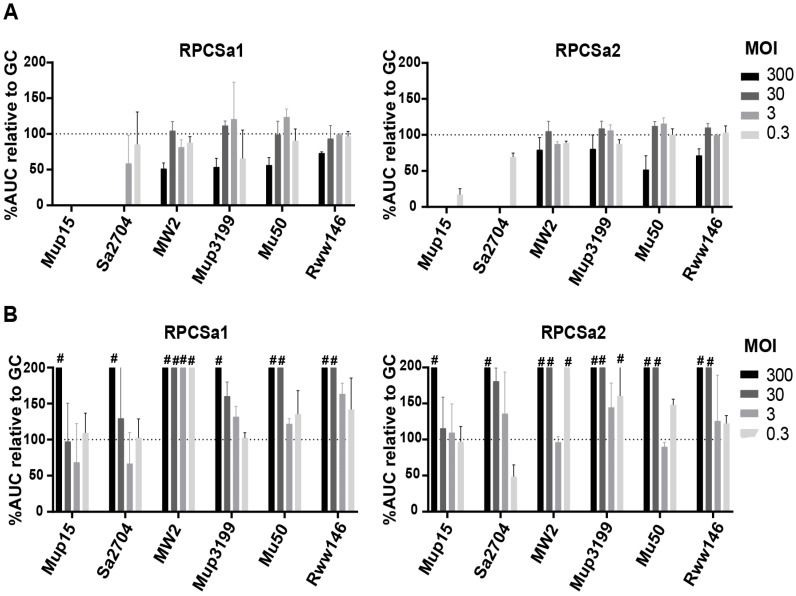
Phage susceptibility in TSB compared to 82% human serum. Microcalorimetry was used to measure bacterial heat production after incubation of isolated single phages with two highly sensitive strains (Mup15 and SA2704), two moderate sensitive strains (MW2 and Mup3199) and two non-sensitive strains (Mu50 and Rww146) of *S. aureus*. Heat flow was measured for twenty hours in either (**A**) TSB or (**B**) 82% human serum and is shown as a percentage of the area under the curve of the growth control (no phages added). ^#^ For clarity, values above 200% are not shown. All conditions were tested in three independent experiments.

Contrary to the phage cocktails, RPCSa1 and RPCSa2 could be purified and produced in high concentrations. They were therefore used to determine phage susceptibility of *S. aureus* in human serum at higher MOI than tested using the phage cocktails (Figure 7). Two phage-sensitive (Mup15 and SA2704), two moderately sensitive (MW2 and Mup3199) and two resistant strains (Mu50 and Rww146) of *S. aureus* were selected. High concentrations of RPCSa1 or RPCSa2 were added in 82% TSB (Figure 7A) or human serum (Figure 7B). While phage susceptibility in TSB was similar to that observed in the OD assay, susceptibility of all strains including the highly phage-sensitive strains was completely absent in human serum even at MOI 300 (Figure 7B). Bacterial growth in phage-treated conditions even exceeded the bacterial growth control in serum, especially at the highest concentrations of phages. Further investigation showed this was caused by the presence of TSB in the phage solutions, as adding nutrients together with the phages resulted in increased bacterial growth (Supplementary Materials Figure S3).

## 4. Discussion

For phage therapy to be successful, the susceptibility of the bacterial strain causing the infection will first need to be determined prior to the administration of phages. For this purpose, in vitro assays are currently used, which are less time consuming and less expensive compared to animal experiments. However, current in vitro results do not always correlate with in vivo data, with differences in phage susceptibility potentially being caused by differences in the microenvironment between in vivo and in vitro assays. Therefore, in this publication, three in vitro phage susceptibility assays were used to investigate the impact of experimental conditions on the susceptibility of clinical *S. aureus* isolates to phages. Our results showed a clear difference in phage susceptibility between the assays using bacterial culture media and an assay using 82% serum.

In both the OD assay and spot test, three commercial phage cocktails and the single phages used, there was a broad host range against clinical *S. aureus* strains, despite the genetic variation between the *S. aureus* isolates evaluated. This is in line with previous in vitro data showing a broad host range for these cocktails [67,68]. These results can be explained by the presence of *Kayvirus* phages in each cocktail and in the similarity of the single phages to phage K, the species type of this genus. *Kayvirus* phages are often used in phage cocktails because their genomes contain only a limited number of restriction sites, thereby limiting the recognition and destruction of their DNA by restriction enzymes of bacterial RM-systems [30,69,70]. In addition, these phages bind to WTA, the only known phage-receptor of *S. aureus*. However, while most phages bind to specific WTA glycan modifications (that can differ between bacterial strains and under different environmental conditions), *Kayvirus* phages bind to the backbone of WTA and are therefore not affected by these modifications [12,14,69,70,71].

Despite these phage characteristics, not all *S. aureus* strains were lysed in our experiments. Interestingly, the genomes of the *S. aureus* strains that showed low phage susceptibility were not genetically closely related to each other, as shown by the phylogenetic analysis. Therefore, no clear link between phage susceptibility and the genetic background of our *S. aureus* strains could be made. Moreover, the evaluation of known phage-resistance genes in the genome could not explain the differences of phage susceptibility of the isolates either. For example, although Mup3199 was resistant to the phage cocktails tested, no known phage-resistance genes were found. In contrast, R5 is very phage-sensitive despite the presence of multiple phage-resistant genes. This is consistent with the observation of Moller et al. that phage susceptibility relies on both host factors, most of which are still unknown, and phage-specific factors. The lack of a clear relation between the genetic background of isolates and their phage susceptibility highlights the importance of susceptibility testing prior to treatment with phages [48].

Comparison of the spot test and OD assay did not reveal identical phage susceptibility patterns for the *S. aureus* strains tested. For example, strain M116 was susceptible to GPC and RPC in the spot test but not in the OD assay. This supports previous observations that the spot test might give an overestimation of phage susceptibility [64]. Discrepancies between the two conventional assays were also seen for the single phages but only at the highest MOI, where three *S. aureus* strains that were not susceptible in the spot test showed a decrease in growth in the OD assay. However, this high MOI might not be realistic for in vivo use because bacteriophages do not accumulate well in all tissues and are cleared from the blood both passively by the spleen and liver and actively by the immune system [72]. Some of these discrepancies could be contributed to a lower accuracy of the spot test as compared to ‘efficiency of plating’ (EOP) [10,64]. However, while EOP did match the OD results for some strains where the spot test did not, differences between all conventional assay still remained for other strains. Next to discrepancies between these conventional assays, which could lead to under- or over-estimation of phage susceptibility, these assays lack resemblance with the in vivo microenvironment.

In contrast, MC allowed the determination of phage susceptibility in human serum, despite the presence of bacterial aggregation, showing a drastic decrease in phage susceptibility to both the phage cocktails and single phages when compared to TSB. However, it should be noted that due to the unknown content of the commercial phage cocktails used, we were not able to concentrate them without the risk of selecting for specific phages [66]. As a result, the cocktails were not tested at the same high MOI that was used for the single phages. Nevertheless, these results confirm previous observations by Shinde et al. (2022), who showed reduced phage infectivity under similar conditions. However, in the study of Shinde et al., bacterial aggregation was observed to be a limitation of the test [65]. The lack of phage susceptibility in serum could, in part, be due to a reduced growth rate of *S. aureus* in human serum, resulting in reduced phage propagation [73,74]. However, growth controls still exhibited significant signal, albeit lower than in TSB. Previous studies have shown direct binding of antibodies to phages, thereby directly preventing phage binding directly; moreover, competition between phages and antibodies directed to WTA could indirectly prevent phage binding [20,21,73,75,76].

In this study, we highlight the importance of experimental conditions on the phage susceptibility of *S. aureus*. The lack of phage susceptibility in human serum could explain the discrepancy between in vitro results obtained using conventional assays and in vivo data from clinical phage therapy trials. For example, the intravenous administration of AB-SA01 (a cocktail containing *Kayvirus* phages) resulted in a response rate of only 62%, despite the spot test indicating complete in vitro phage susceptibility of the infecting bacterial strain [77]. So even though it is a commonly used assay, the spot test might not be able to fully predict phage susceptibility in vivo. Although exhibiting a reduced response rate, these results do suggest a contribution of AB-SA1 in potentially helping to neutralize this infection, even during intravenous administration. Whether this result is actually due to lysis of the bacteria by phages, stimulation of the immune system or other processes needs to be further investigated.

Here, we propose the use of MC testing in combination with current conventional assays, for more accurate in vitro phage selection for phage therapy. This is due to the assay’s better resemblance to the microenvironment encountered by bacteria and phages in vivo. In the future, MC could be used to determine phage susceptibility in other media resembling in vivo environments. For example, it has already been shown to be a valuable tool for phage susceptibility determination in urine [61]. MC might even lead to a novel form of personalized therapy, in which both the infectious strain and the bodily fluids of a patient can be used for bacteriophage selection. Nevertheless, future studies which correlate MC data with data of clinical trials are needed to close the gap between in vitro phage selection and in vivo phage susceptibility. Together, this research might ultimately contribute to improvement of phage selection for phage therapy.

## Figures and Tables

**Table 2:** Overview of the presence of phage resistance genes in the *S. aureus* strains.

	MSSA Strains					MRSA Strains					Lab Strains		
Genes *	Mup15	Mup3199	Mup2723	Mup2396	SA2704	MW2	Mu50	SaC042W	M116	Rww146	RN4220	R5	
*isdB*			+ ^#^			+	+			+	+	+	Moller et al. (2021) [48]
*mrpF*			+			+	+			+	+	+	
*relA*			+			+	+			+	+	+	
*tarP*										+			
*phoR*			+			+	+			+	+	+	
*tarS*			+			+	+			+	+	+	
*tarM*			+		+	+	+				+	+	
*fmtC*			+			+	+			+	+	+	
*tagH*			+			+	+			+	+	+	
*trpA*			+			+	+			+	+	+	
*tarJ*			+			+	+			+	+	+	
*sodM2*			+			+	+			+	+	+	
*hsdR*			+	+	+	+			+	+	+	+	
*hsdM*	+		+	+		+	+			+	+	+	
*sau3AIR*	+												
*gajA*									+				PADLOC [51]
*gajB*									+				
*drt4*													
*thsB*										+			
*thsA*										+			
*lmuA*					+								
*lmuB*					+								
*avs2*						+					+		

* Genes described by Moller et al. (2021) [48] examined using BLASTn and phage defense systems found by the online tool PADLOC [51]. Genes included are involved in WTA biosynthesis (*tarJ*, *tagH)*, modification (*tarP*, *tarS*, *tarM*) and degradation (*phoR*); restriction-methylation (*hsdR*, *hsdM* and *sau3AIR*); abortive infection (*thsA* and *thsB*); cell surface stress tolerance (*sodM*), surface charge (*fmtC*, *mrpF*) and surface occlusion (*isdB*); bacterial metabolism (*relA*, *trpA*); phage resistance mechanisms that are not yet fully understood (*gajA*, *gajB*, *lmuA*, *lmuB*, *drt4* and *avs2*) [13,48,63] ^#^ Genes that are present genes are indicated with a ‘+’.

**Table 3:** Susceptibility of ten clinical and two laboratory *S. aureus* strains to three phage cocktails and two single phages as determined by the spot test.

		*RPC*	*GPC*	*INT*	*RPCSa2*	*RPCSa1*
*MSSA* *strains*	Mup15	++ ^#^	++	++	++++	++++
	Mup3199				+++	+++
	Mup2723	++	++	++	++++	++++
	Mup2396					
	SA2704	+	+	+	+++	+++
*MRSA* *strains*	MW2		+	+	+++	+++
	Mu50					
	SaC042W	++	++		+++	+++
	M116	++	+			+++
	Rww146					
*Lab* *strains*	RN4220	++	++		+++	++++
	R5	+	+	++	+++	+++

^#^ Lysis of bacterial strains is indicated with a ‘+’ for individual plaques found at a phage dilution of 10^−1^ to 10^−2^, ‘++’ for plaques found at a dilution of 10^−3^ to 10^−4^, ‘+++’ for plaques found at dilution 10^−6^ to 10^−7^ and ‘++++’ for plaques found at a dilution of 10^−8^. Empty cells indicate no observed lysis.

**Table 4:** Discrepancies between the spot test (ST) and OD assay were assessed using ‘efficiency of plating’ (EOP).

	*RPC*			*GPC*			*INT*			*RPCSa2*			*RPCSa1*		
	ST	OD	EOP	ST	OD	EOP	ST	OD	EOP	ST	OD	EOP	ST	OD	EOP
Mup3199	-^#^	+	+	-	+	+	-	+	+						
Mup2396	-	+	-							-	+	+	-	+	+
MW2	-	+	+				+	-	+						
Mu50	-	+	-	-	+	-				-	+	+	-	+	+
SaC042W	+	-	+				-	+	+						
M116	+	-	+	+	-	+				-	+	+			
Rww146	-	+	-							-	+	-	-	+	-
RN4220							-	+	+						

^#^ Lysis of bacterial strains are indicated with a ‘+’ and no observed lysis is indicated with ‘-‘. When no discrepancies between the spot test and OD assay were observed, EOP was not performed (empty cells).

## Data Availability

The nucleotide sequences generated in this study have been deposited in NCBI with accession numbers OP352909 and OP352910 (tail sheath proteins). Accession numbers for whole genome sequences of the sequenced *S. aureus* isolates are provided in Table 1.

## Supplementary Materials

The following supporting information can be downloaded at https://www.mdpi.com/article/10.3390/v15010014/s1: Table S1: Overview of bacteriophage genera present in the three commercially available phage cocktails; Figure S1: Representative curves of the optical density (OD) over time; Figure S2: Area under the curve of the optical density of *S. aureus* isolates incubated with phage cocktails in TSB; Figure S3: Microcalorimetry in 82% serum in presence of tryptic soy broth (TSB).
