# Supplementary material for: Microcalorimetry: A Novel Application to Measure In Vitro Phage Susceptibility of Staphylococcus aureus in Human Serum
Source: Viruses. 2022 Dec 20;15(1):14. doi: 10.3390/v15010014 (PMC9865112; doi:10.3390/v15010014)
Supplement: Supplementary file 1 [file viruses-15-00014-s001.zip › viruses-2036252-supplementary.pdf]

viruses MDPI

Supplementary Materials

Table S1. Overview of bacteriophage genera present in the three commercially available phage cocktails Russian Pyofag (RPC), Georgian Pyofag (GPC) and Intestifag (INT).

	Bacteriophage genus	*% mapped reads*	*Bacterial host (genus)*[#]
RPC	*Efquatrovirus*	23	*Enterococcus*
	Saphexavirus	17	*Escherichia*
	Kayvirus	**14**	***Staphyloccocus***
	Kochikohdavirus	14	*Enterococcus*
	Phapecoctavirus	7	*Escherichia*
	Panjvirus	4	*Salmonella*
	Tequatrovirus	3	*Escherichia*
	Teseptimavirus	3	*Escherichia, Shigella, Yersinia, Salmonella*
	Phikzvirus	2	*Pseudomonas*
	Phikmvvirus	2	*Pseudomonas*
	Mosigvirus	1	*Escherichia, Shigella*
	Kayfunavirus	1	*Escherichia, Citrobacter, Cronobacter, Enterobacter, Salmonella, Shigella*
	Other[*]	9	-
GPC	*Saphexavirus*	20	*Escherichia*
	Phapecoctavirus	14	*Escherichia*
	Teseptimavirus	10	*Escherichia, Shigella, Yersinia, Salmonella*
	Novosibovirus	9	*Proteus*
	Acadevirus	9	*Proteus*
	Kayfunavirus	9	*Escherichia, Citrobacter, Cronobacter, Enterobacter, Salmonella, Shigella*
	Przondovirus	7	*Klebsiella, Escherichia,*
	Teetrevirus	5	*Serratia, Citrobacter, Enterobacter, Klebsiella, Escherichia, Salmonella, Shigella, Yersinia, Leclercia, Pantoea*
	Webervirus	2	*Klebsiella, Stenotrophomonas*
	Drulisvirus	2	*Klebsiella, Shigella, Escherichia*
	Kayvirus	**1**	***Staphyloccocus***
	Other[*]	22	**-**
INT	*Saphexavirus*	36	*Escherichia*
	Kayvirus	**11**	***Staphyloccocus***
	Berlinvirus	9	*Escherichia, Shigella, Salmonella, Yersinia, Enterobacter, Pectobacterium*
	Kochikohdavirus	8	*Enterococcus*
	Phapecoctavirus	5	*Escherichia*
	Panjvirus	4	*Salmonella*
	Tequatrovirus	4	*Escherichia*
	Phikmvvirus	3	*Pseudomonas*
	Phikzvirus	2	*Pseudomonas*
	Seunavirus	2	*Escherichia, Salmonella, Cronobacter*
	Jersey virus	2	*Salmonella, Escherichia, Xanthomonas*
	Teseptimavirus	2	*Escherichia, Shigella, Yersinia, Salmonella*
	Phikzvirus	2	*Pseudomonas*
	Efquatrovirus	1	*Enterococcus*
	Mosigvirus	1	*Escherichia, Shigella*
	Other[*]	8	-

[*]Genera to which ≥1% of the reads are mapped.

#Based on nomenclature of bacteriophage species belonging to a genus according to the NCBI taxonomy database.

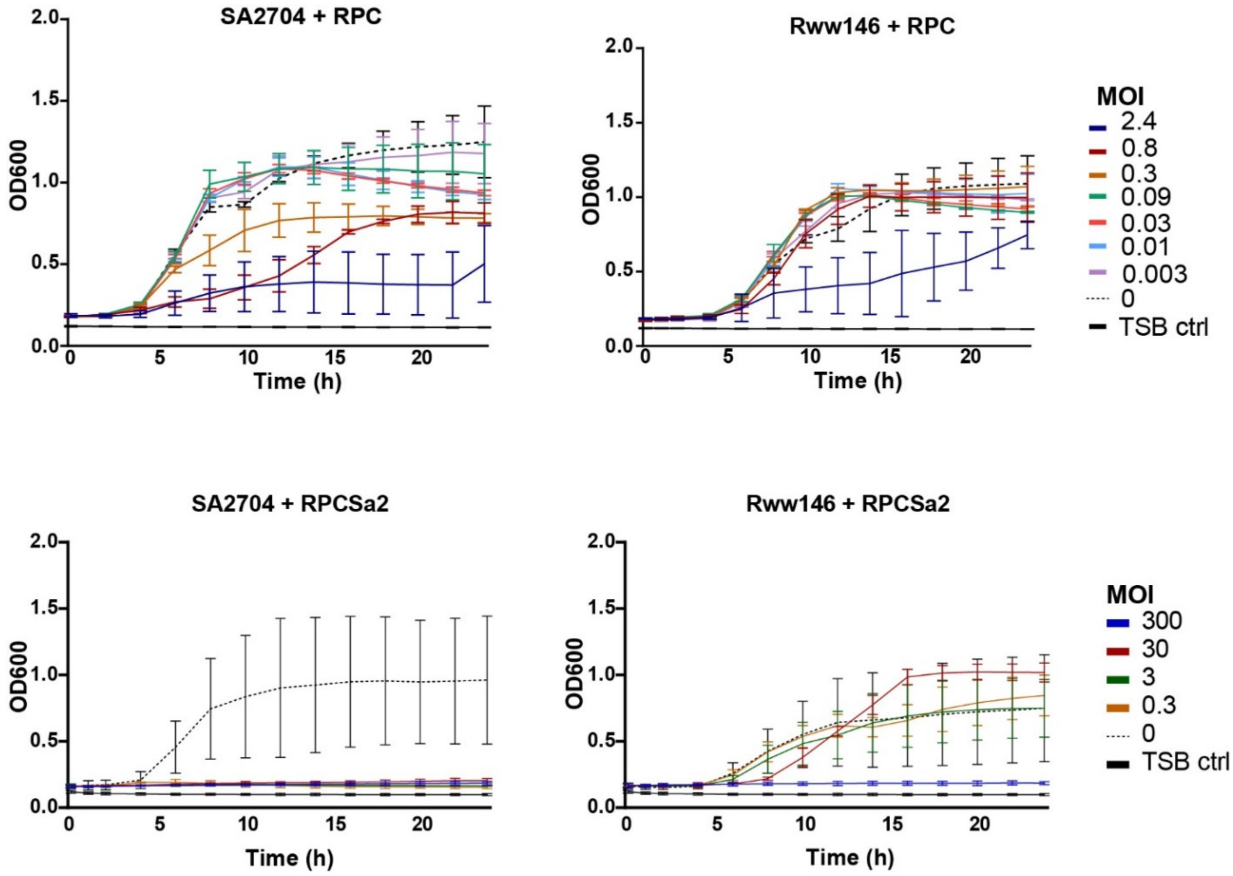

Figure S1. Representative curves of the optical density (OD) over time. All *S. aureus* strains were incubated with different dilutions of (a) the phage cocktails (here represented by the RPC) or (b) the single phages. Here an MSSA and MRSA strain are depicted as representatives for the panel of *S. aureus* strains used. The OD was measured every ten minutes for 24 hours in three independent experiments.

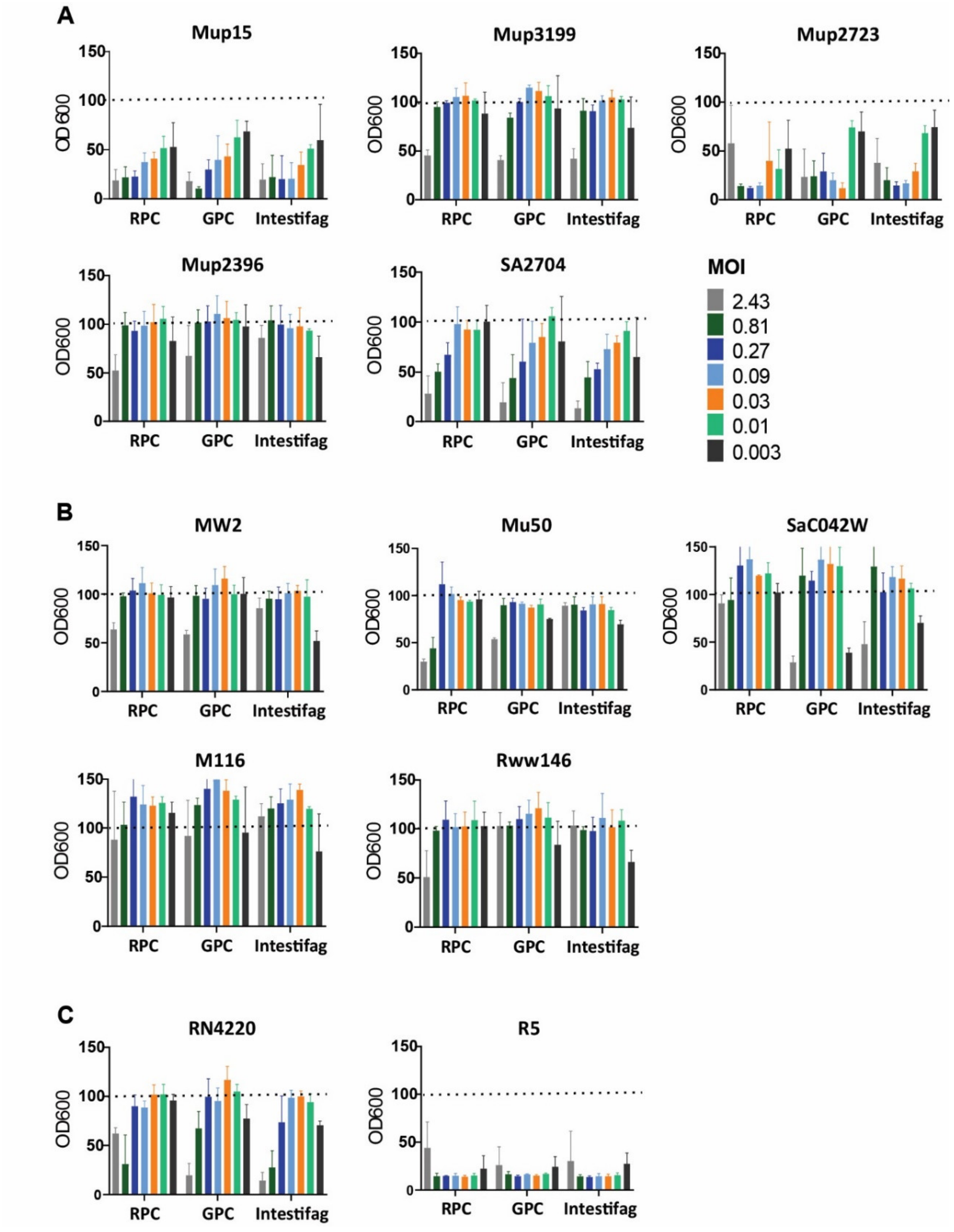

Figure S2. Area under the curve of the optical density of S. aureus isolates incubated with phage cocktails in TSB. The OD of (**a**) five MSSA strains, (**b**) five MRSA strains and (**c**) two laboratory strains inoculated with phages at different MOI was measured for 24 hours. Three-fold dilutions of the phage cocktails were tested. The OD over time (24h) is depicted as percentage of area under the curve (AUC) compared to the growth control, which was set at 100%. All conditions were repeated in three independent experiments.

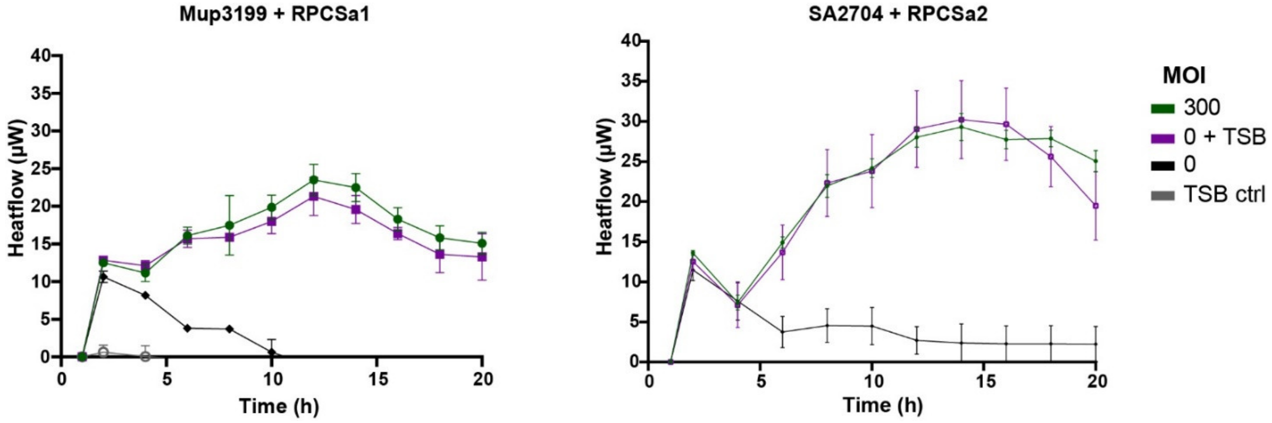

Figure S3. Microcalorimetry in 82% serum in presence of tryptic soy broth (TSB). Heat flow was measured over 20 hours in three independent assays. Heat flow of the bacteria incubated with the highest MOI of the single phages (propagated in TSB) (green) overlaps with the heat flow of the growth control in which the same volume of TSB (without phages) is added to the bacteria (purple).
